# Taurine priming improves redox balance, osmotic adjustment, and nutrient acquisition to lessen phytotoxic effects of neutral and alkaline salts on pea (*Pisum sativum* L.)

**DOI:** 10.1080/15592324.2025.2480224

**Published:** 2025-03-25

**Authors:** Umer Farooq, Ayesha Rehman, Muhammad Arslan Ashraf, Rizwan Rasheed, Mudassar Shahid, Shafaqat Ali, Pallab K. Sarker

**Affiliations:** a Department of Botany, Government College University Faisalabad, Faisalabad, Pakistan; b Department of Pharmaceutics, College of Pharmacy, King Saud University, Riyadh, Saudi Arabia; c Department of Environmental Sciences and Engineering, Government College University Faisalabad, Faisalabad, Pakistan; d Department of Biological Sciences and Technology, China Medical University, Taichung, Taiwan; e Environmental Studies Department, University of California Santa Cruz, Santa Cruz, CA, USA

**Keywords:** Oxidative damage, ROS homeostasis, signaling molecules, osmotic adjustment, secondary metabolism

## Abstract

Taurine (TAR) intricately mediates a plethora of physiological processes. This investigation aimed to elucidate the impact of TAR (50, 100, 150, and 200 mg L^−1^) seed priming on redox homeostasis, glutathione metabolism, photosynthetic efficiency, osmotic adjustment and nutrient acquisition in pea plants subjected to 100 mm salinity of neutral (NaCl and Na_2_SO_4_) and alkaline (Na_2_CO_3_) salts. Salinity diminished growth, chlorophyll, and photosynthetic efficiency alongside a concurrent rise in reactive oxygen species (ROS), lipid peroxidation, and relative membrane permeability. Seed priming with 150 mg L^−1^ TAR efficiently enhanced growth by reducing oxidative damage to plants under salinity. Taurine enhanced leaf relative water content through osmotic adjustment facilitated by the induced accumulation of proline, glycine betaine, soluble sugars, and total free amino acids. Taurine increased the levels of antioxidant compounds and the activities of enzymes, which assisted in the detoxification of ROS and methylglyoxal. Taurine maintained chlorophyll integrity and enhanced photosynthetic efficiency by alleviating oxidative stress. Taurine diminished Na content, which improved the acquisition of essential nutrients under the salinity of neutral and alkaline salts. The results suggest that TAR has a potential role in maintaining ion homeostasis, crucial for enhancing pea tolerance to salt stress.

## Introduction

Global climate change threatens agriculture by intensifying abiotic stresses such as extreme temperatures, drought, and soil salinity, thereby affecting crop productivity and food security. Anthropogenic activities have accelerated glacier melting, rising sea levels, and extreme weather events while altering precipitation patterns, leading to increased soil salinization and expanding salt-affected areas.^1,2^ Saline and alkaline conditions commonly coexist in soils with different proportions of neutral and alkaline salts. Soil salinization occurs due to the accumulation of salts, primarily sodium (Na^+^) and chloride (Cl^−^) ions, which raise the salinity of the soil solution, impair water uptake, and induce osmotic stress in plants.^3,4^ Saline and alkaline conditions disrupt vital physiological processes such as photosynthesis, transpiration, respiration, enzyme activity, and nutrient balance.^5^ Alkaline salts are particularly detrimental, as they raise soil pH levels, which can significantly hinder nutrient absorption, thus exacerbating stress on plant growth.^6^ Salt stress affects over 33% of irrigated land globally and 20% of the total cultivated area.^7^ In Pakistan, approximately 4.5 million hectares are impacted by soil salinity.^8^

Salinity markedly increased the generation of reactive oxygen species (ROS), including superoxide (O₂•^−^), singlet oxygen (^1^O₂), hydrogen peroxide (H₂O₂), and hydroxyl radicals (•OH).^9^ The oxidative stress due to induced ROS production damages the integrity of the cell membrane under salinity.^10^ Salinity enhances lipoxygenase (LOX) activity, which catalyzes the oxidation of membrane polyunsaturated fatty acids, leading to lipid peroxidation in membranes.^11,12^ To mitigate ROS damage, plants activate a range of antioxidant defense systems, which include both enzymatic and non-enzymatic components, to effectively scavenge ROS and limit lipid peroxidation.^13,14^ Superoxide dismutase (SOD) catalyzes the conversion of O₂•^−^ into H₂O₂, playing a pivotal role in ROS detoxification.^15^ Catalase (CAT) further dismutates H₂O₂ into water, primarily within peroxisomes.^16^ Ascorbate peroxidase (APX), located in mitochondria, chloroplasts, cytosol, and peroxisomes, utilizes ascorbate as an electron donor to convert H₂O₂ to water.^14^ Methylglyoxal (MG), a highly reactive and toxic compound, accumulates in plants under various abiotic stress conditions.^17^ Glutathione (GSH) compounds are crucial in detoxifying MG by converting it into less harmful compounds, thus protecting plants from MG-induced stress.^18^

Pea (*Pisum sativum* L.), a member of the *Leguminosae* family, is a legume crop with significant nutritional and economic value.^19^ Peas are also rich in essential minerals like iron and zinc, vitamins, and carbohydrates. Limited research has focused on enhancing pea plant tolerance to salinity of neutral and alkaline salts.^20^ The application of different chemical treatments, including plant hormones, amino acids, and nutrients, has gained considerable attention as an efficient strategy to mitigate abiotic stress.^21^ Taurine (TAR), a non-protein amino acid, has notable cytoprotective functions owing to its antioxidant properties.^22^ Although recent studies on TAR effectiveness in overcoming environmental abiotic stresses in plants are limited, evidence suggests that TAR application enhances tolerance in clover plants to manganese stress,^23^ and improves tolerance to nickel stress in bottle gourd.^24^ However, there remains a significant gap in understanding the physiological capacity of TAR priming to alleviate neutral and alkaline stress in pea plants. It is hypothesized that TAR seed priming could reduce the negative impacts of saline and alkaline stress on pea plants. This study aims to investigate the potential of TAR in enhancing growth, redox balance, osmotic adjustment, glutathione content, nutrient acquisition, and accumulation of signaling molecules like nitric oxide and hydrogen sulfide in pea plants under salinity of neutral and alkaline salts.

## Materials and methods

### Plant material, growth conditions, and treatment

The experiment was executed in the Botanic Garden, Government College University Faisalabad. One pea cultivar, Meteor, was used in the present research to evaluate the efficacy of taurine to improve tolerance against neutral and alkaline salts. Sodium hypochlorite solution (1.0%) was used to sterilize pea (*Pisum sativum* L.) seeds for 10 minutes. The seeds were then rinsed five times with distilled water and primed for 18 h in TAR (50, 100, 150, and 200 mg L^‒1^) following.^25^ Ten seeds were planted in a pot filled with 8 kg of soil. The soil utilized in the experiment was sandy loam with 8.4 pH and 2.17 dS m^‒1^ electrical conductivity. Throughout the investigation, the average temperature was 24°C, the relative humidity was 43%, and the photosynthetically active radiation (PAR) was 1213 µmol m^‒2^ s^‒1^. After two weeks of sowing, the plants received diammonium phosphate, potassium sulfate, and urea (60, 130, and 30 kg ha^‒1^) as phosphorus (P), potassium (K), and nitrogen (N) sources, respectively.^26^ Three plants were retained, and the remaining plants were manually removed and macerated in the same pot. Thirty-day-old plants were subjected to 100 mm of each salt (NaCl, Na_2_SO_4_, and Na_2_CO_3_). 60-day plants were harvested to examine changes in several physiological and biochemical characteristics. The seed priming treatments under non-saline and alkaline salts (NaCl, Na_2_SO_4,_ and Na_2_CO_3_) are listed below:

1. Unprimed
2. TAR 50 mg L^−1^
3. TAR 100 mg L^‒1^
4. TAR 150 mg L^‒1^
5. TAR 200 mg L^‒1^

### Growth parameters

Shoot fresh and dry biomass and leaf area were quantified from the harvested plant material. Following the measurement of fresh plant biomass, plants were desiccated in an oven at 65°C to determine dry biomass. The method described by Gardner et al.^27^ was employed to assess the leaf area of pea plants.

### Chlorophyll florescence

Chlorophyll fluorescence parameters were measured using OPTISciences, OS5P, following the methodology described by Mousavi et al.^28^

### Leaf pigments

Fresh leaf tissue was utilized to determine photosynthetic leaf pigments, including chlorophyll *a*, *b*, total chlorophyll, and carotenoids, following the method described by Lichtenthaler.^29^

### Antioxidant pigments

Sarker and obe^30^ protocol was applied to determine the levels of β-cyanin and β-xanthine. The supernatant was obtained by homogenizing leaf tissue in 80% methanol. Jensen et al.^31^ method was employed to determine β-carotene from the aqueous methanol extract.

### Non-enzymatic antioxidants

Anthocyanin content in fresh leaf tissue was quantified utilizing the assay described by Julkunen et al.^32^ From fresh leaf material, ascorbic acid was determined employing the method established by Mukherjee and choudhuri.^33^ In fresh leaf tissue, the phenolic content was assessed using the protocol outlined by Wolfe.^34^ The assay delineated by Zhishen et al.^35^ was employed to quantify the flavonoid content of fresh leaf tissue.

### Osmolytes

Total soluble sugars were measured from ethanolic extract of fresh leaf material.^36^ Nelson.^37^ approach was used to quantify reducing sugars from ethanolic extract of fresh leaf tissue. The calculation of non-reducing sugars involved subtracting reducing sugars from the total soluble sugars, as prescribed by Loomis and Shull.^38^ The assessment of total free amino acids in fresh leaf tissue extracts utilized the K-P buffer extraction method described by Hamilton.^39^ The proline content of leaves was measured using the Bates approach.^40^ The determination of glycine betaine in fresh leaf tissue employed the methodology outlined by Grieve and Grattan.^41^

### Hydrogen sulphide (H_2_S) and nitric oxide (NO)

The H_2_S measurement was conducted in accordance with the Nashef et al.^42^ After pulverizing 0.5 g of leaf tissue in 10 mL of potassium phosphate buffer containing 50 mm, the supernatant was extracted by centrifugation. Next, 20 µL of 20 mm 5,5-dithiobis-2-nitrobenzoic acid, 0.188 mL of 50 mm potassium phosphate buffer (pH 7.5), and 1.7 mL of deionized water were combined with a 0.1 mL aliquot of the supernatant in test tubes. At 412 nm, optical density was then measured.

For the purpose of estimating nitric oxide, the procedure described by Zhou was followed.^43^ 0.5 g of leaf tissue was homogenized in 10 mL of cooled 50 mm acetate buffer, and the supernatant was collected. Following the addition of 100 mg of charcoal, the supernatant was filtered. The filtrate was subsequently combined with 1 mL of Griess reagent. Upon incubation at room temperature for 20 minutes, the optical density was determined at 540 nm.

### Enzyme assays

To measure total soluble proteins (TSP) and enzyme activity, 0.5 g of fresh plant tissue was homogenized in 10 mL of 50 mm potassium buffer (pH 7.5). The homogenate was centrifuged at 12,000 rpm for 10 minutes at 4°C, and the supernatant was collected for subsequent TSP and enzyme assays. The total soluble protein content was determined using the Bradford method.^44^

The method outlined by Giannopolitis and Ries^45^ was used to measure the activity of SOD in the reaction mixture by tracking the photochemical inhibition of nitroblue tetrazolium chloride and recording the optical density at 560 nm.

To measure peroxidase (POD) activity, a reaction mixture was prepared by adding 0.1 mL of enzyme extract, 10 mm H_2_O_2_, and 20 mm guaiacol to a total volume of 3 mL. The increase in absorbance at 470 nm was recorded following the method of Chance and Maehly.^46^

Catalase (CAT) activity was assessed using the protocol described by Chance and Maehly.^46^ The reaction mixture comprised 20 mm H_2_O_2_, 100 µL of enzyme extract, and 0.5 M potassium phosphate buffer (pH 7.0). The decline in absorbance at 240 nm was monitored to measure the enzyme activity.

We measured APX activity according to the guidelines set forth by Nakano and Asada.^47^ 2400 µL of 50 mm potassium phosphate buffer, 300 µL of ascorbic acid (0.5 mm), 0.1 mL of Na-EDTA (0.1 mm), and 0.1 mm H2O2 made up the reaction mixture. At 290 nm, a decrease in absorbance was noted.

### Glutathione contents

GSH and GSSG levels were measured using the procedure outlined by Hasanuzzaman et al.^48^ 10 mL of 5% metaphosphoric acid and 1 mm EDTA were used to finely grind fresh leaf tissue. The supernatant was recovered after centrifugation and combined with 5,5-dithiobis (2-nitrobenzoic acid) and 50 mm potassium phosphate buffer at pH 7.5. Subsequently, the optical density of the reaction mixture was measured at 412 nm. The optical density was remeasured at 412 nm after 2-vinylpyridine was added to eliminate GSH in order to determine GSSG.

### Oxidants and degree of lipid peroxidation

The analytical procedure employed in this study was developed by Velikova et al.^49^ whose methodology was utilized to quantify the concentration of hydrogen peroxide (H_2_O_2_) in fresh leaf material (0.25 g).

The levels of superoxide radicals (O_2_^•‒^) were assessed in fresh leaf tissue samples (0.5 g) using the methodology developed by Yang et al.^50^

The methylglyoxal (MG) content of fresh plant tissue samples (0.5 g) was quantified utilizing the method described by Nahar et al.^51^ The absorption was measured using a spectrophotometer at the recommended wavelength of 288 nm.

To determine the concentration of malondialdehyde (MDA), a 0.5 g sample of fresh leaf tissue was homogenized in 6% trichloroacetic acid. The resultant supernatant was subsequently subjected to a reaction with 5% thiobarbituric acid, following the protocol delineated by Cakmak and Horst.^52^

Lipoxygenase (LOX) activity was quantified utilizing the protocol described by Doderer et al.^53^ which involved combining 0.1 mL of enzyme extract with 0.5 mL of linoleic acid substrate solution and 2.4 mL of distilled water, followed by measurement of the absorbance at 234 nm.

Fresh leaf tissue samples (0.5 g) were excised and immersed in 10 mL of distilled water for 24 hours, after which the initial electrical conductivity (EC1) was measured. The samples were subsequently subjected to a temperature treatment, comprising a 1-hour water bath followed by a 20-minute cooling period at 25°C, before the final electrical conductivity (EC2) was determined, as described by Lutts et al.^54^

### DPPH• scavenging activity

DPPH• radical scavenging activity was evaluated utilizing the procedure described by Sarker and Oba,^30^ employing fresh samples ground in acetone.

### Nitrate reductase activity

The method outlined by Joyia et al.^55^ was utilized to assess nitrate reductase activity. The reaction mixture consisted of the enzyme extract, 0.02% 1-naphthyl-ethylenediamine dihydrochloride solution, and 1% sulfanilamide solution. Absorbance was measured at 542 nm following incubation of the mixture for 30 minutes at 32°C.

### Ions analysis

The elemental analysis of plant dry matter, comprising leaves and roots, was conducted through acid digestion following the protocol established by Allen et al.^56^ Phosphorus content was assessed using the methodology described by Jackson.^57^ A flame photometer (Sherwood, model 410, UK) was employed to quantify the levels of sodium (Na), potassium (K), and calcium (Ca) in the samples.

### Statistical analysis

A completely randomized design with factorial arrangements was implemented for the experiment. Each treatment was replicated four times. XLSTAT software (v. 2017.3, Paris, France) was used to compute analysis of variance. Tukey’s HSD test at *p* ≤ 0.05 determined significant differences between treatment means.

## Results

### Growth attributes

Salinity of NaCl, Na_2_CO_3_, and Na_2_SO_4_ caused a notable decline in various growth characteristics. Maximal reduction in shoot length (22.18%, 32.09%, and 27.37%), root length (32%, 52.61%, 37.58%), shoot fresh weight (16.48%, 36.7%, and 39.70%), root fresh weight (42.13%, 47.97%, and 48.53%), shoot dry weight (31.40%, 39.97%, and 5.36%) and root dry weight (28.01%, 35.24%, and 30.57%) was noted in plants stressed with NaCl, Na_2_SO_4_, and Na_2_CO_3_ salts, respectively. Taurine (TAR) priming notably enhanced plant growth under NaCl, Na_2_CO_3_, and Na_2_SO_4_ toxicity. Seed priming using TAR (50, 100, 150, and 200 mg L^‒1^) led to considerable enhancement in growth parameters. TAR (150 mg L^‒1^) showed a higher increase in shoot length (28.77%), root length (34.44%), shoot dry weight (28.78%) and root dry weight (34.02%) under Na_2_CO_3_ salt stress, while shoot fresh weight (36.38%) root fresh weight (49.61%) demonstrate maximal surge under Na_2_SO_4_ stress, respectively (Table 1).

**Table 1.** Effect of taurine (TAR) on shoot length, root length, shoot fresh weight, root fresh weight, shoot dry weight, root dry weight of pea plants grown under soil spiked with 100 mm sodium chloride (NaCl), 100 mm sodium carbonate (Na_2_CO_3_), 100 mm sodium sulfate Na_2_SO_4_ toxicity. Values given in the table are the means ± standard error of four replicates (*n* = 4). The means with different superscript letters are significantly different from each other according to Tukey’s honestly significant difference (HSD) at *p* ≤ 0.05.

Stress	Treatment	Shoot length (cm)	Root length (cm)	Shoot fresh weight (g)	Root fresh weight (g)	Shoot dry weight (g)	Root dry weight (g)
Control	**Unprimed**	53.46 ± 1.893^AB^	34.93 ± 1.114^B^	14.91 ± 0.487^AB^	9.968 ± 0.390^A^	4.098 ± 0.126^B^	1.873 ± 0.085^B^
	**TAR 50 mg L**^**−1**^	53.50 ± 0.714^AB^	35.51 ± 1.894^AB^	14.34 ± 0.916^ABC^	9.990 ± 0.232^A^	4.067 ± 0.019^B^	1.638 ± 0.084^C^
	**TAR 100 mg L**^**−1**^	54.64 ± 1.683^A^	37.57 ± 0.579^AB^	15.52 ± 0.445^AB^	10.12 ± 0.329^A^	4.453 ± 0.031^A^	1.908 ± 0.079^AB^
	**TAR 150 mg L**^**−1**^	54.59 ± 1.219^A^	38.25 ± 1.652^A^	15.71 ± 0.687^A^	10.40 ± 0.472^A^	4.470 ± 0.049^A^	2.005 ± 0.012^A^
	**TAR 200 mg L**^**−1**^	54.99 ± 1.638^BC^	36.06 ± 0.413^AB^	14.99 ± 0.873^A^	9.995 ± 0.335^A^	3.973 ± 0.200^B^	1.995 ± 0.031^AB^
100 mm NaCl	**Unprimed**	41.60 ± 2.257^FGH^	23.75 ± 1.315^EFG^	12.45 ± 0.398^DE^	5.767 ± 0.431^GH^	2.811 ± 0.149^FG^	1.348 ± 0.051^FG^
	**TAR 50 mg L**^**−1**^	42.94 ± 1.122^E-H^	24.91 ± 1.373^DEF^	12.97 ± 0.527^CD^	6.613 ± 0.382^D-G^	3.128 ± 0.028^DE^	1.540 ± 0.018^CDE^
	**TAR 100 mg L**^**−1**^	43.25 ± 1.377^E-H^	27.00 ± 1.472^CD^	13.47 ± 0.402^BCD^	7.525 ± 0.148^BCD^	3.335 ± 0.046^CD^	1.553 ± 0.013^CDE^
	**TAR 150 mg L**^**−1**^	46.25 ± 1.548^C-F^	29.00 ± 0.707^C^	14.54 ± 0.436^AB^	7.920 ± 0.208^B^	3.460 ± 0.095^C^	1.615 ± 0.024^C^
	**TAR 200 mg L**^**−1**^	43.68 ± 1.604^D-H^	26.75 ± 0.854^CD^	13.47 ± 0.398^BCD^	7.743 ± 0.243^BC^	3.248 ± 0.035^CD^	1.665 ± 0.026^C^
100 mm Na_2_CO_3_	**Unprimed**	36.30 ± 1.286^I^	16.55 ± 1.355^K^	9.440 ± 0.724^IJ^	5.186 ± 0.505^H^	2.460 ± 0.124^HI^	1.213 ± 0.024^H^
	**TAR 50 mg L**^**−1**^	39.27 ± 2.358^HI^	18.00 ± 0.408^JK^	9.660 ± 0.193^HIJ^	6.216 ± 0.195^EFG^	2.680 ± 0.100^GH^	1.238 ± 0.013^GH^
	**TAR 100 mg L**^**−1**^	40.10 ± 1.480^GHI^	19.75 ± 0.479^HIJ^	10.21 ± 0.408^G-J^	6.337 ± 0.295^EFG^	2.895 ± 0.048^EFG^	1.440 ± 0.058^EF^
	**TAR 150 mg L**^**−1**^	46.75 ± 1.315^CDE^	22.25 ± 0.629^FGH^	12.19 ± 0.270^DEF^	7.663 ± 0.286^BC^	3.168 ± 0.100^CDE^	1.625 ± 0.062^C^
	**TAR 200 mg L**^**−1**^	39.92 ± 1.885^GHI^	19.18 ± 0.910^IJK^	11.11 ± 0.750^E-H^	6.802 ± 0.267^C-F^	3.085 ± 0.036^DEF^	1.545 ± 0.038^CDE^
100 mm Na_2_SO_4_	**Unprimed**	38.83 ± 0.990^HI^	21.80 ± 0.294^GHI^	8.990 ± 0.602^J^	5.130 ± 0.594^H^	2.033 ± 0.118^J^	1.300 ± 0.020^GH^
	**TAR 50 mg L**^**−1**^	44.75 ± 2.780^C-G^	23.33 ± 0.879^EFG^	10.94 ± 0.244^E-I^	5.965 ± 0.282^FGH^	2.305 ± 0.029^IJ^	1.365 ± 0.01^FG^
	**TAR 100 mg L**^**−1**^	48.50 ± 2.661^CD^	23.50 ± 0.645^EFG^	11.26 ± 0.355^EFG^	6.938 ± 0.164^CDE^	2.397 ± 0.147^HI^	1.318 ± 0.019^FGH^
	**TAR 150 mg L**^**−1**^	49.00 ± 2.449^BC^	26.00 ± 1.080^DE^	12.26 ± 0.460^DEF^	7.675 ± 0.122^BC^	2.470 ± 0.062^HI^	1.578 ± 0.035^CD^
	**TAR 200 mg L**^**−1**^	36.00 ± 0.707^I^	22.00 ± 1.080^F-I^	10.77 ± 0.452^F-I^	7.110 ± 0.365^B-E^	2.060 ± 0.229^J^	1.448 ± 0.075^DEF^

### Chlorophyll florescence

The findings indicated a significant decrease in quantum efficiency‒ΦPSII (21.53%, 24.80%, and 23.92%), quenching coefficient‒qP (29.22%, 37.24%, and 27.63%), maximum efficiency of photosystem II‒Fv/Fm (25.66%, 29.47%, and 21.40%), and quantum yield‒qL (10.23%, 21.59%, and 23.30%) in pea plants. In contrast, a notable increase (46.76%, 47.98%, and 41.11%) in non-photochemical quenching coefficient (ΦNPQ) value was observed under NaCl, Na_2_CO_3,_ and Na_2_SO_4_ stress, respectively. Furthermore, seed priming with TAR (150 mg L‒1) exhibited the most significant enhancement in ΦPSII (28.29%) and qP (43.61%) under Na_2_CO_3_, Fv/Fm (18.79%) under NaCl, and qL (17.63%) under Na_2_SO_4_ stress (Table 2).

**Table 2.** Effect of taurine (TAR) on quantum efficiency (ΦPSII), quenching coefficient (qP), maximum efficiency of photosystem-ii (Fv/Fm), quantum yield (qL), and non-photochemical quenching coefficient (ΦNPQ) of pea plants grown under soil spiked with 100 mm sodium chloride (NaCl), 100 mm sodium carbonate (Na_2_CO_3_), 100 mm sodium sulfate Na_2_SO_4_ toxicity. Values given in the table are the means ± standard error of four replicates (*n* = 4). The means with different superscript letters are significantly different from each other according to Tukey’s honestly significant difference (HSD) at *p* ≤ 0.05.

Stress	Treatment	ΦPSII	qP	Fv/Fm	qL	ΦNPQ
Control	**Unprimed**	1.076 ± 0.012^AB^	0.963 ± 0.019^A^	0.891 ± 0.009^A^	0.738 ± 0.017^AB^	0.474 ± 0.027^D^
	**TAR 50 mg L**^**−1**^	1.071 ± 0.022^AB^	0.930 ± 0.042^ABC^	0.892 ± 0.017^A^	0.698 ± 0.003^ABC^	0.515 ± 0.031^BCD^
	**TAR 100 mg L**^**−1**^	1.091 ± 0.005^A^	0.939 ± 0.017^AB^	0.892 ± 0.028^A^	0.729 ± 0.014^AB^	0.538 ± 0.021^A-D^
	**TAR 150 mg L**^**−1**^	1.120 ± 0.026^A^	0.968 ± 0.016^A^	0.902 ± 0.029^A^	0.772 ± 0.018^A^	0.513 ± 0.020^BCD^
	**TAR 200 mg L**^**−1**^	1.072 ± 0.030^AB^	0.940 ± 0.025^AB^	0.848 ± 0.023^AB^	0.645 ± 0.026^ABC^	0.506 ± 0.038^CD^
100 mm NaCl	**Unprimed**	0.844 ± 0.031^FG^	0.682 ± 0.009^GH^	0.662 ± 0.034^GH^	0.662 ± 0.020^ABC^	0.696 ± 0.007^A^
	**TAR 50 mg L**^**−1**^	0.855 ± 0.032^EFG^	0.688 ± 0.012^G^	0.713 ± 0.025^D-G^	0.689 ± 0.032^ABC^	0.648 ± 0.039^ABC^
	**TAR 100 mg L**^**−1**^	0.893 ± 0.067^D-G^	0.747 ± 0.011^EFG^	0.680 ± 0.013^FGH^	0.686 ± 0.011^ABC^	0.653 ± 0.018^ABC^
	**TAR 150 mg L**^**−1**^	0.981 ± 0.024^BCD^	0.859 ± 0.041^CD^	0.786 ± 0.024^B-E^	0.729 ± 0.024^AB^	0.593 ± 0.015^A-D^
	**TAR 200 mg L**^**−1**^	0.883 ± 0.024^D-G^	0.682 ± 0.005^GH^	0.704 ± 0.031^E-H^	0.624 ± 0.038^BC^	0.644 ± 0.047^ABC^
100 mm Na_2_CO_3_	**Unprimed**	0.809 ± 0.011^G^	0.604 ± 0.025^H^	0.628 ± 0.032^H^	0.578 ± 0.038^C^	0.702 ± 0.010^A^
	**TAR 50 mg L**^**−1**^	0.888 ± 0.031^D-G^	0.726 ± 0.054^EFG^	0.647 ± 0.042^GH^	0.623 ± 0.036^BC^	0.647 ± 0.029^ABC^
	**TAR 100 mg L**^**−1**^	0.945 ± 0.026^CDE^	0.789 ± 0.050^DE^	0.669 ± 0.032^GH^	0.633 ± 0.021^ABC^	0.633 ± 0.021^ABC^
	**TAR 150 mg L**^**−1**^	1.038 ± 0.011^ABC^	0.868 ± 0.012^BCD^	0.716 ± 0.028^D-G^	0.641 ± 0.018^ABC^	0.525 ± 0.036^BCD^
	**TAR 200 mg L**^**−1**^	0.818 ± 0.035^G^	0.707 ± 0.017^FG^	0.712 ± 0.035^D-G^	0.626 ± 0.033^BC^	0.626 ± 0.033^A-D^
100 mm Na_2_SO_4_	**Unprimed**	0.819 ± 0.027^G^	0.697 ± 0.024^FG^	0.700 ± 0.041^FGH^	0.566 ± 0.023^C^	0.669 ± 0.014^AB^
	**TAR 50 mg L**^**−1**^	0.876 ± 0.056^EFG^	0.709 ± 0.044^FG^	0.692 ± 0.030^FGH^	0.574 ± 0.041^C^	0.653 ± 0.046^ABC^
	**TAR 100 mg L**^**−1**^	0.923 ± 0.051^DEF^	0.775 ± 0.023^EF^	0.794 ± 0.029^BCD^	0.612 ± 0.041^BC^	0.599 ± 0.040^A-D^
	**TAR 150 mg L**^**−1**^	1.041 ± 0.014^ABC^	0.894 ± 0.021^ABC^	0.831 ± 0.016^ABC^	0.594 ± 0.033^BC^	0.568 ± 0.031^A-D^
	**TAR 200 mg L**^**−1**^	0.903 ± 0.061^D-G^	0.800 ± 0.022^DE^	0.761 ± 0.035^C-F^	0.665 ± 0.024^ABC^	0.639 ± 0.032^ABC^

### Leaf area, leaf relative water content, and photosynthetic pigments

Pea plants exhibited significant reductions in leaf area (40.47%, 45.93%, and 43.07%), leaf relative water content (16.09%, 29.22%, 24.23%), chlorophyll a (18.85%, 25.73%, and 19.33%), chlorophyll b (16.53%, 47.05%, and 29.70%), total chlorophyll (18.22%, 31.46%, and 22.12%), and carotenoids (41.08%, 34.90%, and 19.06%) under NaCl, Na_2_CO_3_, and Na_2_SO_4_ stress, respectively. Furthermore, TAR priming (50, 100, 150, 200 mg L^‒1^) significantly ameliorated salt stress effects on leaf area, LRWC, and photosynthetic pigments in pea plants. Taurine (150 mg L^‒1^) increased leaf area (60.58%), LRWC, (23.38%), chlorophyll *b* (68.79%), total chlorophyll (28.89%) under Na_2_CO_3_ stress, conversely chlorophyll *a* display higher increment (24.60%) under NaCl and carotenoids (66.06%) under Na_2_SO_4_ stress (Table 3).

**Table 3.** Effect of taurine (TAR) on leaf area, leaf relative water content, chlorophyll *a* chlorophyll *b*, total chlorophyll, and carotenoids in pea plant grown under soil spiked with 100 mm sodium chloride (NaCl), 100 mm sodium carbonate (Na_2_CO_3_), 100 mm sodium sulfate Na_2_SO_4_ toxicity. Values given in the table are the means ± standard error of four replicates (*n* = 4). The means with different superscript letters are significantly different from each other according to Tukey’s honestly significant difference (HSD) at *p* ≤ 0.05.

Stress	Treatment	Leaf Area (cm^2^)	Leaf Relative Water Content (%)	Chlorophyll *a* (mg g^−1^ FW)	Chlorophyll *b* (mg g^−1^ FW)	Total Chlorophyll (mg g^−1^ FW)	Carotenoids (mg g^−1^ FW)
Control	**Unprimed**	9.525 ± 0.258^BC^	79.42 ± 1.338^BC^	1.774 ± 0.152^BCD^	0.654 ± 0.012^CDE^	2.428 ± 0.155^CD^	0.021 ± 0.001^GH^
	**TAR 50 mg L**^**−1**^	9.586 ± 0.422^B^	83.20 ± 1.351^AB^	1.967 ± 0.110^AB^	0.677 ± 0.049^BCD^	2.644 ± 0.065^ABC^	0.022 ± 0.001^GH^
	**TAR 100 mg L**^**−1**^	11.20 ± 0.904^A^	90.52 ± 3.503^A^	2.062 ± 0.096^A^	0.779 ± 0.032^A^	2.841 ± 0.085^A^	0.019 ± 0.001^H^
	**TAR 150 mg L**^**−1**^	11.32 ± 0.448^A^	85.24 ± 8.098^AB^	2.143 ± 0.015^A^	0.743 ± 0.017^AB^	2.886 ± 0.030^A^	0.021 ± 0.002^GH^
	**TAR 200 mg L**^**−1**^	8.704 ± 0.822^BCD^	84.31 ± 2.085^AB^	1.960 ± 0.032^AB^	0.785 ± 0.003^A^	2.745 ± 0.033^AB^	0.025 ± 0.003^FGH^
100 mm NaCl	**Unprimed**	5.670 ± 0.556^GHI^	66.64 ± 4.092^EFG^	1.440 ± 0.109^FGH^	0.546 ± 0.019^FGH^	1.985 ± 0.115^FGH^	0.029 ± 0.002^DEF^
	**TAR 50 mg L**^**−1**^	6.163 ± 0.230^F-I^	70.21 ± 3.691^DEF^	1.479 ± 0.090^FGH^	0.558 ± 0.017^EFG^	2.036 ± 0.094^EFG^	0.030 ± 0.002^DEF^
	**TAR 100 mg L**^**−1**^	8.100 ± 0.576^B-E^	69.65 ± 6.089^DEF^	1.751 ± 0.100^BCD^	0.689 ± 0.004^BC^	2.440 ± 0.103^CD^	0.031 ± 0.002^CDE^
	**TAR 150 mg L**^**−1**^	8.031 ± 0.648^CDE^	76.71 ± 2.164^BCD^	1.798 ± 0.144^BC^	0.735 ± 0.012^AB^	2.533 ± 0.136^BC^	0.037 ± 0.002^ABC^
	**TAR 200 mg L**^**−1**^	6.840 ± 0.231^E-H^	71.54 ± 3.734^CDE^	1.504 ± 0.122^E-H^	0.613 ± 0.029^DEF^	2.116 ± 0.142^EFG^	0.027 ± 0.004^EFG^
100 mm Na_2_CO_3_	**Unprimed**	5.150 ± 0.295^I^	56.21 ± 1.072^H^	1.318 ± 0.015^H^	0.346 ± 0.010^J^	1.664 ± 0.015^I^	0.028 ± 0.001^EF^
	**TAR 50 mg L**^**−1**^	6.064 ± 0.576^F-I^	58.87 ± 1.176^GH^	1.325 ± 0.025^GH^	0.452 ± 0.024^I^	1.777 ± 0.014^HI^	0.025 ± 0.002^FGH^
	**TAR 100 mg L**^**−1**^	7.168 ± 0.857^EFG^	63.80 ± 0.822^E-H^	1.498 ± 0.026^E-H^	0.536 ± 0.046^GH^	2.034 ± 0.054^EFG^	0.028 ± 0.003^EF^
	**TAR 150 mg L**^**−1**^	8.270 ± 0.264^B-E^	69.36 ± 1.504^DEF^	1.560 ± 0.017^D-G^	0.585 ± 0.058^EFG^	2.145 ± 0.048^EF^	0.033 ± 0.002^A-D^
	**TAR 200 mg L**^**−1**^	8.155 ± 0.595^B-E^	63.82 ± 1.941^E-H^	1.448 ± 0.036^FGH^	0.544 ± 0.026^FGH^	1.991 ± 0.059^FGH^	0.027 ± 0.001^EF^
100 mm Na_2_SO_4_	**Unprimed**	5.423 ± 0.278^HI^	60.18 ± 0.819^GH^	1.431 ± 0.079^FGH^	0.460 ± 0.016^I^	1.891 ± 0.089^FGI^	0.025 ± 0.001^EFG^
	**TAR 50 mg L**^**−1**^	6.245 ± 0.510^F-I^	62.70 ± 1.023^GH^	1.537 ± 0.130^D-H^	0.478 ± 0.011^I^	2.015 ± 0.139^FGH^	0.028 ± 0.003^EF^
	**TAR 100 mg L**^**−1**^	7.560 ± 0.427^DEF^	64.38 ± 1.372^E-H^	1.578 ± 0.036^C-F^	0.512 ± 0.011^HI^	2.090 ± 0.040^EFG^	0.035 ± 0.001^A-D^
	**TAR 150 mg L**^**−1**^	8.290 ± 0.660^B-E^	65.23 ± 1.459^EFG^	1.693 ± 0.006^CDE^	0.584 ± 0.027^EFG^	2.276 ± 0.023^DE^	0.041 ± 0.001^A^
	**TAR 200 mg L**^**−1**^	7.568 ± 0.406^DEF^	69.26 ± 1.328^DEF^	1.628 ± 0.033^C-F^	0.513 ± 0.014^GHI^	2.140 ± 0.034^EF^	0.038 ± 0.003^AB^

### Antioxidants pigments

β-carotene (18.72%, 38.74%, and 38.28%) decreased significantly in pea plants, whereas β-cyanin (55.07%, 47.11%, and 67.17%), β-xanthin (12.53%, 19.72%, and 56.76%), and anthocyanin (22.58%, 142.03%, and 97.53%) exhibited notable increases in pea plants under NaCl, Na_2_CO_3_, and Na_2_SO_4,_ respectively. However, exogenous TAR (50, 100, 150, and 200 mg L-1) significantly increased antioxidant pigments under salt stress conditions. Furthermore, 150 mg L-1 TAR exhibited a more pronounced increase in β-carotene (48.12%) under Na_2_SO_4_ stress, β-cyanin (38.66%) under NaCl stress, and β-xanthin (56.73%) under Na_2_CO_3_ stress (Table 4).

**Table 4.** Effect of taurine (TAR) on β-carotene, β-cyanin, β-xanthin, anthocyanin, ascorbic acid, and phenolics in pea plant grown under soil spiked with 100 mm sodium chloride (NaCl), 100 mm sodium carbonate (Na_2_CO_3_), 100 mm sodium sulfate Na_2_SO_4_ toxicity. Values given in the table are the means ± standard error of four replicates (*n* = 4). The means with different superscript letters are significantly different from each other according to Tukey’s honestly significant difference (HSD) at *p* ≤ 0.05.

Stress	Treatment	β-carotene (ng g^−1^ FW)	β-cyanin (ng g^−1^ FW)	β-xanthin (ng g^−1^ FW)	Anthocyanin (mg g^−1^ FW)	Ascorbic acid (mg g^−1^ FW)	Phenolics (mg g^−1^ FW)
Control	**Unprimed**	0.054 ± 0.001^AB^	10.81 ± 0.951^J^	15.30 ± 1.216^HI^	1.279 ± 0.274^I^	6.010 ± 0.412^H^	21.45 ± 1.782^i^
	**TAR 50 mg L**^**−1**^	0.051 ± 0.002^ABC^	10.67 ± 0.820^J^	16.25 ± 0. 691^HI^	1.336 ± 0.124^I^	6.852 ± 0.392^GH^	23.06 ± 0.703^HI^
	**TAR 100 mg L**^**−1**^	0.047 ± 0.001^B-F^	11.97 ± 0.583^J^	17.63 ± 0.718^GHI^	1.636 ± 0.144^HI^	6.778 ± 0.332^GH^	27.10 ± 1.596^GH^
	**TAR 150 mg L**^**−1**^	0.049 ± 0.003^A-D^	12.40 ± 0.676^IJ^	18.51 ± 1.767^E-I^	1.848 ± 0.024^GH^	7.917 ± 0.403^FG^	28.25 ± 0.663^FGH^
	**TAR 200 mg L**^**−1**^	0.043 ± 0.003^D-G^	13.81 ± 0.821^HIJ^	15.18 ± 1.682^I^	1.575 ± 0.103^HI^	6.050 ± 0.290^H^	18.18 ± 1.949^I^
100 mm NaCl	**Unprimed**	0.044 ± 0.002^D-G^	16.77 ± 0.269^FGH^	17.21 ± 0.877^GHI^	1.568 ± 0.069^HI^	8.968 ± 0.366^EF^	31.42 ± 1.047^D-G^
	**TAR 50 mg L**^**−1**^	0.047 ± 0.003^C-F^	20.40 ± 0.299^C-F^	18.69 ± 1.770^E-H^	1.958 ± 0.082^GH^	9.427 ± 0.479^DE^	36.21 ± 1.506^CD^
	**TAR 100 mg L**^**−1**^	0.049 ± 0.002^A-F^	20.86 ± 0.633^C-F^	21.54 ± 0.949^C-F^	2.103 ± 0.023^FG^	10.56 ± 0.107^BCD^	40.38 ± 2.965^BC^
	**TAR 150 mg L**^**−1**^	0.053 ± 0.002^AB^	23.25 ± 0.850^ABC^	22.36 ± 0.686^CD^	2.493 ± 0.027^DEF^	11.16 ± 0.526^ABC^	43.07 ± 0.875^B^
	**TAR 200 mg L**^**−1**^	0.036 ± 0.003^IJ^	22.21 ± 0.503^ABC^	21.88 ± 0.961^CDE^	2385 ± 0.174^EF^	9.132 ± 0.382^EF^	29.31 ± 0.394^EFG^
100 mm Na_2_CO_3_	**Unprimed**	0.033 ± 0.002^J^	15.91 ± 0.219^GHI^	18.31 ± 1.349^FI^	3.096 ± 0.288^ABC^	10.54 ± 0.359^EF^	28.02 ± 2.199^FGH^
	**TAR 50 mg L**^**−1**^	0.035 ± 0.003^IJ^	17.56 ± 1.045^E-H^	22.54 ± 1.135^CD^	3.139 ± 0.202^ABC^	10.16 ± 0.341^CDE^	33.75 ± 3.199^DE^
	**TAR 100 mg L**^**−1**^	0.042 ± 0.001^F-I^	19.72 ± 0.635^C-F^	26.60 ± 0.465^AB^	3.353 ± 0.138^AB^	11.02 ± 0.434^ABC^	35.94 ± 1.316^CD^
	**TAR 150 mg L**^**−1**^	0.045 ± 0.003^C-F^	21.81 ± 0.874^A-D^	28.70 ± 1.385^A^	3.478 ± 0.012^A^	11.88 ± 0.570^A^	44.03 ± 1.126^B^
	**TAR 200 mg L**^**−1**^	0.038 ± 0.003^HIJ^	19.82 ± 0.612^C-F^	19.95 ± 1.176^D-G^	3.200 ± 0.023^ABC^	11.50 ± 0.347^AB^	41.09 ± 2.826^BC^
100 mm Na_2_SO_4_	**Unprimed**	0.033 ± 0.001^J^	18.08 ± 1.086^D-G^	23.98 ± 1.624^BC^	2.527 ± 0.120^DE^	10.09 ± 0.576^CDE^	33.00 ± 2.555^DEF^
	**TAR 50 mg L**^**−1**^	0.037 ± 0.001^HIJ^	20.85 ± 0.719^B-E^	26.54 ± 0.398^AB^	2.594 ± 0.185^DE^	10.68 ± 0.582^A-D^	39.18 ± 1.047^BC^
	**TAR 100 mg L**^**−1**^	0.041 ± 0.002^F-I^	21.98 ± 0.285^A-D^	28.69 ± 1.502^A^	2.819 ± 0.235^CD^	10.24 ± 0.585^B-E^	43.02 ± 2.251^B^
	**TAR 150 mg L**^**−1**^	0.049 ± 0.002^A-E^	24.66 ± 0.589^A^	29.92 ± 1.051^A^	3.040 ± 0.037^BC^	11.88 ± 0.596^A^	51.58 ± 2.007^A^
	**TAR 200 mg L**^**−1**^	0.054 ± 0.002^A^	24.20 ± 1.015^AB^	27.64 ± 1.298^A^	3.024 ± 0.056^BC^	10.96 ± 0.600^ABC^	44.08 ± 2.307^B^

### Non-enzymatic antioxidants

Salinity exhibited a noticeable increase in levels of anthocyanins, ascorbic acid, phenolics, and flavonoids relative to the control. The most significant enhancement was observed in anthocyanins (142.03%) and ascorbic acid (75.36%) under Na_2_CO_3_ stress, while elevated levels of phenolics (55.80%) and flavonoids (44.87%) were noted under Na_2_SO_4_ stress in pea plants. Seed priming with TAR (50, 100, 150, 200 mg L^‒1^) further enhanced non-enzymatic antioxidants. Taurine (150 mg L^‒1^) exhibited maximal accumulation of anthocyanins (58.97%) and ascorbic acid (24.46%) under NaCl stress, whereas phenolics (57.13%) and flavonoids (30.89%) demonstrated maximal accumulation under Na_2_CO_3_ stress relative to unprimed conditions (Table 4-5).

**Table 5.** Effect of taurine (TAR) on flavonoids, total soluble sugar reducing sugar, non reducing sugar, total soluble protein, and total free amino acid in pea plant grown under soil spiked with 100 mm sodium chloride (NaCl), 100 mm sodium carbonate (Na_2_CO_3_), 100 mm sodium sulfate Na_2_SO_4_ toxicity. Values given in the table are means ± standard error of four replicates (*n* = 4). The means with different superscript letters are significantly different from each other according to Tukey’s honestly significant difference (HSD) at *p* ≤ 0.05.

Stress	Treatment	Flavonoids (μg g^−1^ FW)	Total soluble sugar (mg g^−1^ FW)	Reducing sugar (mg g^−1^ FW)	Non reducing sugar (mg g^−1^ FW)	Total soluble protein (mg g^−1^ FW)	Total free amino acid (mg g^−1^ FW)
Control	**Unprimed**	0.036 ± 0.002^I^	16.24 ± 0.737^I^	2.566 ± 0.117^I^	13.67 ± 0.805^H^	2.671 ± 0.116^B^	2.694 ± 0.015^J^
	**TAR 50 mg L**^**−1**^	0.038 ± 0.003^HI^	18.05 ± 1.458^HI^	2.621 ± 0.411^I^	15.43 ± 1.136^FGH^	2.691 ± 0.081^B^	2.887 ± 0.046^J^
	**TAR 100 mg L**^**−1**^	0.045 ± 0.004^E-H^	17.38 ± 1.036^I^	3.181 ± 0.319^GHI^	14.20 ± 0.882^GH^	3.120 ± 0.188^A^	2.911 ± 0.132^J^
	**TAR 150 mg L**^**−1**^	0.041 ± 0.002^GHI^	19.06 ± 1.299^GHI^	2.930 ± 0.215^HI^	16.13 ± 1.147^E-H^	2.916 ± 0.230^AB^	2.965 ± 0.051^J^
	**TAR 200 mg L**^**−1**^	0.045 ± 0.001^E-H^	19.51 ± 1.078^F-I^	3.188 ± 0.310^GHI^	16.32 ± 0.777^E-H^	2.654 ± 0.097^B^	2.696 ± 0.062^J^
100 mm NaCl	**Unprimed**	0.049 ± 0.002^E-F^	19.06 ± 1.350^GHI^	3.738 ± 0.453^E-H^	15.33 ± 1.376^FGH^	1.495 ± 0.029^I^	3.871 ± 0.252^I^
	**TAR 50 mg L**^**−1**^	0.053 ± 0.004^A-D^	21.11 ± 1.887^E-H^	4.153 ± 0.315^B-F^	16.95 ± 1.709^D-G^	2.157 ± 0.071^CD^	4.306 ± 0.038^D-H^
	**TAR 100 mg L**^**−1**^	0.054 ± 0.002^A-D^	24.18 ± 1.597^A-E^	4.519 ± 0.513^A-E^	19.66 ± 1.449^A-D^	2.690 ± 0.063^B^	4.529 ± 0.162^CDE^
	**TAR 150 mg L**^**−1**^	0.058 ± 0.002^AB^	25.43 ± 0.486^A-D^	5.043 ± 0.308^AB^	20.38 ± 0.376^ABC^	2.910 ± 0.063^AB^	4.615 ± 0.164^CDE^
	**TAR 200 mg L**^**−1**^	0.048 ± 0.001^C-F^	18.78 ± 0.907^GHI^	4.691 ± 0.283^A-D^	14.09 ± 0.732^GH^	2.637 ± 0.111^B^	4.224 ± 0.098^E-I^
100 mm Na_2_CO_3_	**Unprimed**	0.043 ± 0.004^F-I^	21.65 ± 1.108^EFG^	3.344 ± 0.351^F-I^	18.30 ± 1.084^C-F^	1.430 ± 0.036^I^	3.942 ± 0.092^HI^
	**TAR 50 mg L**^**−1**^	0.049 ± 0.004^C-F^	23.70 ± 1.482^B-E^	3.999 ± 0.305^C-G^	19.70 ± 1.442^A-D^	1.555 ± 0.047^HI^	4.121 ± 0.109^F-I^
	**TAR 100 mg L**^**−1**^	0.055 ± 0.003^A-D^	21.63 ± 2.073^EFG^	4.908 ± 0.247^ABC^	16.72 ± 2.042^D-H^	1.963 ± 0.174^C-F^	4.355 ± 0.255^D-G^
	**TAR 150 mg L**^**−1**^	0.056 ± 0.002^AB^	26.30 ± 0.864^AB^	5.191 ± 0.298^A^	21.11 ± 1.070^ABC^	2.211 ± 0.161^C^	4.484 ± 0.344^C-F^
	**TAR 200 mg L**^**−1**^	0.054 ± 0.002^A-D^	22.81 ± 0.683^C-F^	3.621 ± 0.499^E-H^	19.19 ± 0.724^B-E^	2.072 ± 0.064^CDE^	4.078 ± 0.018^GHI^
100 mm Na_2_SO_4_	**Unprimed**	0.052 ± 0.003^B-E^	25.74 ± 1.182^A-D^	3.673 ± 0.477^E-H^	22.06 ± 1.353^AB^	1.515 ± 0.024^I^	4.663 ± 0.017^CD^
	**TAR 50 mg L**^**−1**^	0.054 ± 0.002^A-D^	26.40 ± 1.300^AB^	3.801 ± 0.380^D-H^	22.60 ± 1.176^A^	1.638 ± 0.030^GHI^	4.763 ± 0.017^BC^
	**TAR 100 mg L**^**−1**^	0.057 ± 0.003^AB^	26.11 ± 1.033^ABC^	5.015 ± 0.172^AB^	21.10 ± 1.085^ABC^	1.815 ± 0.034^E-H^	5.305 ± 0.085^A^
	**TAR 150 mg L**^**−1**^	0.061 ± 0.003^A^	27.44 ± 0.604^A^	5.123 ± 0.197^A^	22.32 ± 0.759^AB^	1.918 ± 0.046^D-G^	5.405 ± 0.163^A^
	**TAR 200 mg L**^**−1**^	0.051 ± 0.002^B-E^	22.68 ± 0.504^DEF^	3.293 ± 0.160^F-I^	19.39 ± 0.538^A-E^	1.705 ± 0.048^F-I^	5.115 ± 0.015^AB^

### Osmolyte accumulation

Salinity of (NaCl, Na_2_CO_3_, and Na_2_SO_4_) exhibited the highest increase in total soluble sugars (17.40%, 33.31%, and 58.50%), reducing sugars (45.70%, 30.34%, 43.16%), and non-reducing sugars (12.9%, 33.87%, 61.38%) respectively, compared to the control. Furthermore, the administration of TAR (150 mg L^‒1^) as seed priming resulted in the accretion of total soluble sugars (32.98%) and non-reducing sugars (32.98%) under NaCl, respectively. Meanwhile, TAR (150 mg L^‒1^) led to elevated levels of reducing sugars (55.23%) under Na_2_CO_3_ stress compared to unprimed conditions (Table 5).

Salinity, induced by Na_2_CO_3_ salts, notably decreased soluble proteins (46.46%) in pea plants relative to the control. Conversely, TAR (150 mg L^‒1^) display a more significant increase in total soluble proteins (94.64%) under NaCl stress compared to unprimed conditions (Table 5).

A significant increase was observed in total free amino acids (43.70%, 46.34%, and 73.13%), glycine betaine (28.36%, 57.24%, and 47.38%), and proline (42.68%, 66.41%, and 73.13%), under NaCl, Na_2_CO_3_, and Na_2_SO_4_ relative to the control. However, TAR (150 mg L^‒1^) exhibited higher levels of total free amino acids (19.21%) and glycine betaine (29.70%) under NaCl, respectively. TAR (100 mg L‒1) also resulted in higher proline (32.74%) under Na_2_CO_3_ stress (Table 5-6).

**Table 6.** Effect of taurine (TAR) on hydrogen sulfide (H_2_S), nitric oxide (NO), glycine betaine, and proline in pea plant grown under soil spiked with 100 mm sodium chloride (NaCl), 100 mm sodium carbonate (Na_2_CO_3_), 100 mm sodium sulfate Na_2_SO_4_ toxicity. Values given in the table are means ± standard error of four replicates (*n* = 4). The means with different superscript letters are significantly different from each other according to Tukey’s honestly significant difference (HSD) at *p* ≤ 0.05.

Stress	Treatment	Glycine betaine (µg g^−1^ FW)	Proline (µmol g^−1^ FW	H_2_S (µmol g^−1^ FW)	NO (nmol g^−1^ FW)
Control	**Unprimed**	18.43 ± 0.416^I^	1.952 ± 0.173^G^	7.490 ± 0.468^I^	15.30 ± 0.388^I^
	**TAR 50 mg L**^**−1**^	18.50 ± 0.719^I^	1.907 ± 0.042^G^	8. 048 ± 0.904^I^	16.32 ± 0.406^HI^
	**TAR 100 mg L**^**−1**^	21.95 ± 1.028^GH^	2.034 ± 0.099^G^	8.512 ± 0.416^I^	16.41 ± 0.427^HI^
	**TAR 150 mg L**^**−1**^	24.47 ± 1.135^F^	1.880 ± 0.045^G^	7.413 ± 0.513^I^	17.63 ± 0.408^H^
	**TAR 200 mg L**^**−1**^	21.33 ± 0.623^H^	1.762 ± 0.014^G^	7.979 ± 0.672^I^	17.43 ± 0.225^H^
100 mm NaCl	**Unprimed**	23.66 ± 0.813^FG^	2.785 ± 0.076^F^	10.20 ± 0.779^FGH^	22.46 ± 0.341^G^
	**TAR 50 mg L**^**−1**^	27.20 ± 0.479^E^	2.703 ± 0.196^F^	11.65 ± 0.938^C-F^	22.96 ± 0.305^EF^
	**TAR 100 mg L**^**−1**^	28.10 ± 1.419^DE^	2.995 ± 0.102^EF^	10.99 ± 0.567^EFG^	25.24 ± 1.150^CD^
	**TAR 150 mg L**^**−1**^	30.69 ± 0.326^BC^	3.305 ± 0.102^DE^	12.40 ± 0.759^A-E^	27.38 ± 0.516^AB^
	**TAR 200 mg L**^**−1**^	21.91 ± 1.455^GH^	2.779 ± 0.071^F^	9.859 ± 0.330^GH^	21.16 ± 0.738^G^
100 mm Na_2_CO_3_	**Unprimed**	28.99 ± 0.604^CDE^	3.249 ± 0.103^DE^	12.08 ± 0.220^B-E^	23.41 ± 0.556^EF^
	**TAR 50 mg L**^**−1**^	29.91 ± 0.341^BCD^	3.687 ± 0.258^CD^	13.01 ± 1.038^A-D^	23.82 ± 0.721^EF^
	**TAR 100 mg L**^**−1**^	31.39 ± 0.565^B^	4.313 ± 0.037^B^	13.57 ± 0.434^AB^	25.97 ± 0.660^BC^
	**TAR 150 mg L**^**−1**^	35.10 ± 0.836^A^	4.306 ± 0.138^B^	14.04 ± 0.205^A^	27.82 ± 0.646^A^
	**TAR 200 mg L**^**−1**^	29.95 ± 0.604^BCD^	3.608 ± 0.208^CD^	11.37 ± 0.246^D-G^	24.71 ± 0.944^CDE^
100 mm Na_2_SO_4_	**Unprimed**	27.17 ± 0.246^E^	3.807 ± 0.226^C^	11.02 ± 0.799^EFG^	22.95 ± 0.219^EF^
	**TAR 50 mg L**^**−1**^	27.69 ± 0.751^DE^	3.834 ± 0.242^C^	12.46 ± 0.454^A-E^	25.24 ± 0.681^CD^
	**TAR 100 mg L**^**−1**^	32.05 ± 1.039^B^	4.315 ± 0.211^B^	13.26 ± 0.452^ABC^	24.55 ± 0.551^CDE^
	**TAR 150 mg L**^**−1**^	32.08 ± 0.717^B^	4.933 ± 0.183^A^	13.77 ± 0.687^AB^	28.18 ± 0.912^A^
	**TAR 200 mg L**^**−1**^	27.92 ± 0.657^DE^	4.618 ± 0.216^AB^	12.77 ± 0.253^A-D^	25.93 ± 0.857^BC^

### Hydrogen sulfide and nitric oxide

Pea plants treated with Na_2_CO_3_ demonstrated a significant increase in hydrogen sulfide (61.27%) and nitric oxide (53.05%) compared to the control. Moreover, TAR (100 mg L^‒1^) induced a further increase of 21.51% in hydrogen sulfide and 21.88% in nitric oxide under NaCl stress, respectively. Similarly, TAR (150 mg L^‒1^) treatment elicited a maximum level of hydrogen sulfide (24.95%) and nitric oxide (22.79%) under Na_2_SO_4_ stress, respectively (Table 6).

### Antioxidant enzymes

**Figure 1.**
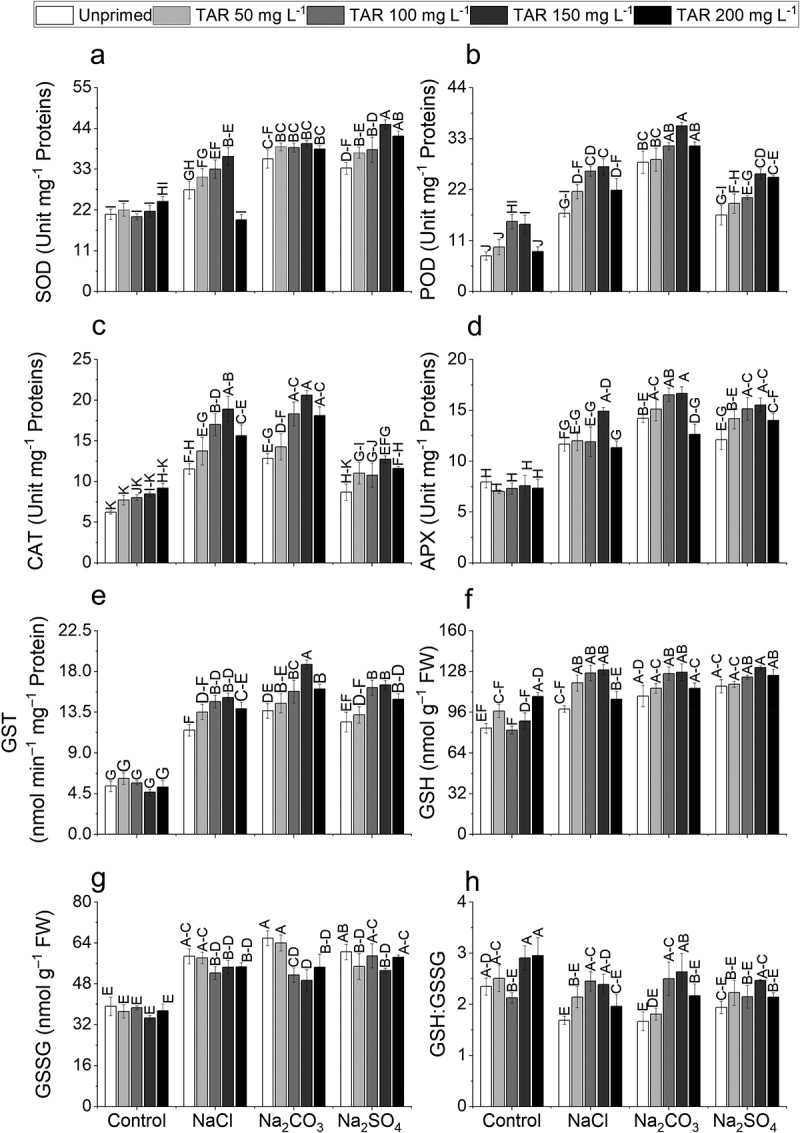
Effect of exogenous taurine on (a) superoxide dismutase activity (SOD), (b) peroxidase activity (POD), (c) catalase activity (CAT), (d) ascorbate peroxidase (APX), (e) glutathione *S*-transferase (GST), (f) glutathione reduced (GSH), (g) glutathione oxidized (GSSG), and (h) their ratio (GSH:GSSG) in pea plants (*Pisum sativum* L.) under NaCl, Na_2_CO_3_, and Na_2_SO_4_ 100 mm each toxicity. Data are means±standard error of four replicates (*n* = 4). The bars represent treatment means followed by different letters are significantly different at 95% confidence level according to Tukey's honestly significant difference (HSD) test.

The activity of antioxidant enzymes increased under (NaCl, Na_2_CO_3_, and Na_2_SO_4_) stress conditions. The most significant rise was observed in the activities of SOD (71.91%), POD (261.34%), CAT (106.4%), APX (78.9%), and GST (155.8%) under Na_2_CO_3_ stress. Taurine (50, 100, 150, 200 mg L-1) enhanced the antioxidant enzyme activity. Furthermore, seed priming with TAR (150 mg L‒1) exhibited the most significant increase in SOD (35.47%) and POD (59.1%) under Na_2_SO_4_ stress, respectively; CAT (63.7%) under NaCl stress, while APX (28.65%) under Na_2_SO_4_ stress. Taurine (150 mg L^‒1^) demonstrated elevated GST activities (37.63%) under Na_2_CO_3_ stress compared to control conditions (Figure 1a-e).

### Glutathione metabolism

Pea plants exhibited a significant increase in GSH (39.43%) under Na_2_SO_4_ stress and GSSG (68.12%) under Na_2_CO_3_ stress, respectively. Additionally, a substantial reduction in the GSH:GSSG ratio (28.91%) was observed under Na_2_CO_3_ stress. Taurine (150 mg L^‒1^) demonstrated maximal increases in GSH (31.22%, 17.16%, and 12.48%) and GSSG (7.23%, 25.12%, and 12.03%) under NaCl, Na_2_CO_3_, and Na_2_SO_4_ stress, respectively. TAR (100 mg L-1) exhibited a higher increase in GSH:GSSG ratio (45.22) under NaCl stress. Additionally, 150 mg L-1 TAR also enhanced GSH:GSSG ratios (57.97%, 27.04%) under Na_2_CO_3_ and Na_2_SO_4_ stress, respectively. (Figure 1f-h).

### Oxidative stress markers

**Figure 2.**
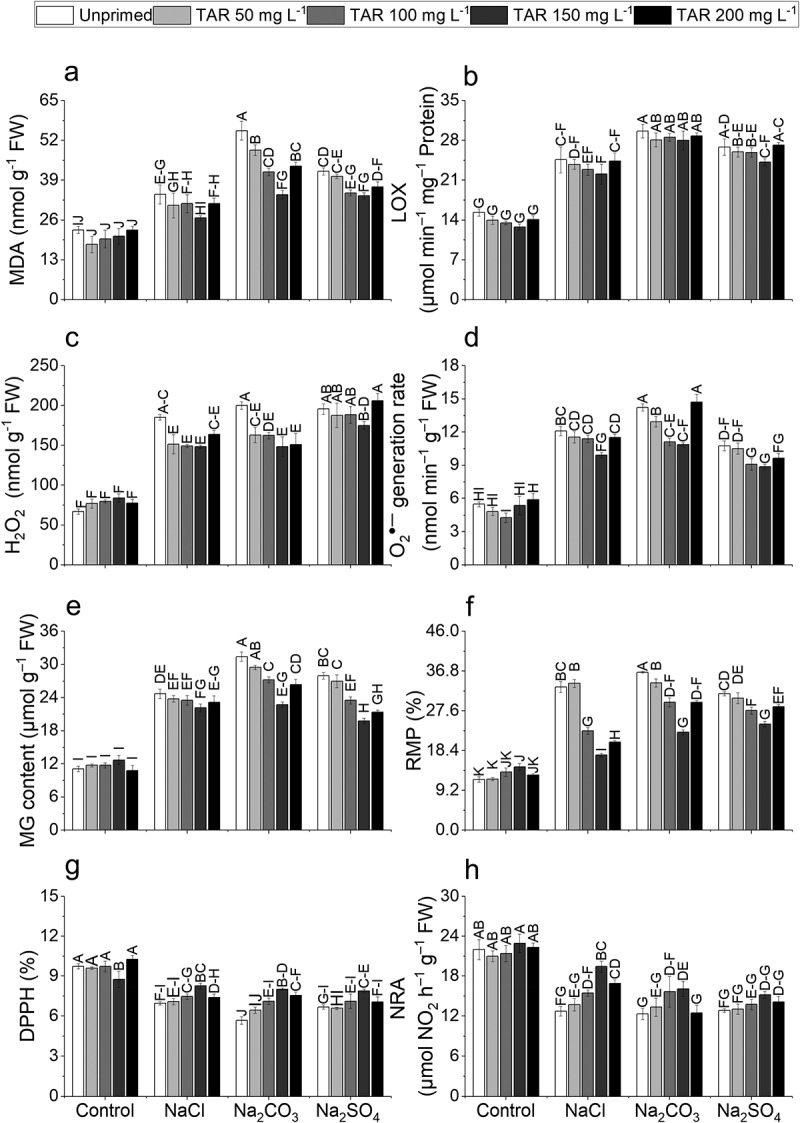
Effect of exogenous taurine on (a) malondialdehyde (MDA), (b) lipoxygenase activity (LOX), (c) hydrogen peroxide (H_2_O_2_), (d) superoxide radical (O_2_^●–^), (e) methylglyoxal (MG), (f) relative membrane permeability (RMP), (g) diphenyl-1-picrylhydrazyl (DPPH), and (h) nitrate reductase (NRA) in pea (*Pisum sativum* L.) plants under NaCl, Na_2_CO_3_, and Na_2_SO_4_ 100 mm each toxicity. Data are means ± standard error of four replicates (*n* = 4). The bars represent treatment means followed by different letters are significantly different at 95% confidence level according to Tukey's honestly significant difference (HSD) test.

Maximal increases (142.36%, 93.36%, 197.29%, 159.87%, 183.21%, and 211.47%) in MDA, LOX, H_2_O_2_, O_2_^•‒^, MG, and RMP, respectively, were observed in plants subjected to Na_2_CO_3_ stress. Furthermore, TAR (50, 100, 150, 200 mg L_–1_) priming significantly reduced oxidative stress markers under NaCl, Na_2_CO_3_, and Na_2_SO_4_ stress conditions compared to unprimed plants. Taurine (150 mg L^‒1^) visibly diminished MDA (37.85%), H_2_O_2_ (25.78%), and O_2_^•‒^ (23.70%) under Na_2_CO_3_, LOX (10.20%) and RMP (47.57%) under NaCl, MG content (29.33%) under Na_2_SO_4_ stress (Figure 2a-f).

### DPPH radical scavenging activity

The exposure to NaCl, Na_2_CO_3_, and Na_2_SO_4_ induced a significant reduction in DPPH levels. Among these stressors, Na_2_CO_3_ exhibited the most pronounced effect, causing a 41.71% decrease in DPPH compared to the control conditions. A significant increase in the DPPH level was observed following seed priming with TAR (50, 100, 150, and 200 mg L^‒1^). Likewise, during Na_2_CO_3_ stress, TAR (150 mg L-1) exhibited higher DPPH values (41.14%) than unprimed samples (Figure 2g).

### Nitrate reductase

Nitrate reductase activity decreased due to the salinity of NaCl, Na_2_CO_3_, and Na_2_SO_4_ salts, with the most pronounced decrease (43.88%) observed under Na_2_CO_3_ compared to the control. In contrast, TAR priming (50 and 100, 150 and 200 mg L^‒1^) notably enhanced NRA activity. Taurine (150 mg L^‒1^) demonstrated higher NRA activity (52.67%) under NaCl stress conditions compared to unprimed plants (Figure 2h).

### Nutrients

**Figure 3.**
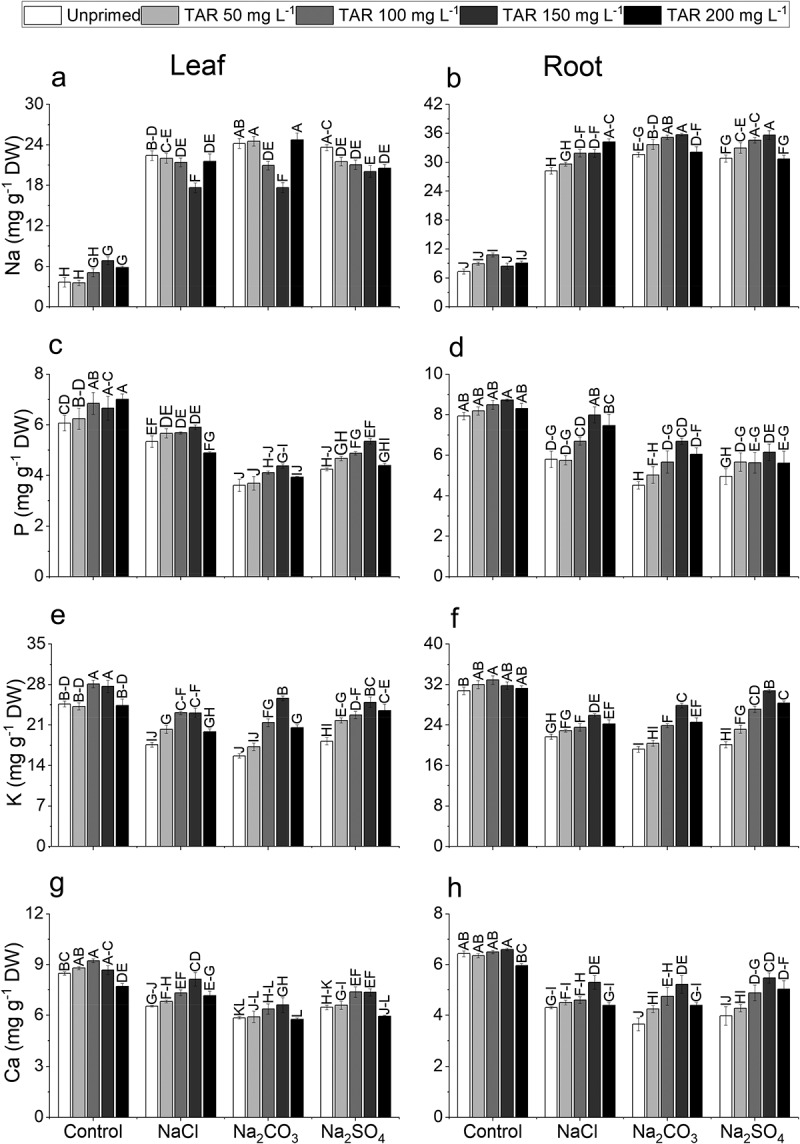
Effect of exogenous taurine on sodium (a-b) leaf and root (Na), (c-d) phosphorus leaf and root (P), (e-f) potassium leaf and root (K), and (g-h) calcium leaf and root (Ca) in pea (*Pisum sativum* L.) plants under NaCl, Na_2_CO_3_, and Na_2_SO_4_ 100 mm each toxicity. Data are means±standard error of four replicates (*n* = 4). The bars represent treatment means followed by different letters are significantly different at 95% confidence level according to Tukey's honestly significant difference (HSD) test.

The nutrient status of plants was significantly altered upon exposure to NaCl, Na_2_CO_3_, and Na_2_SO_4_ salinity. A substantial increase in leaf Na (561.61%) and root Na (330.99%) relative to the control was observed in plants subjected to Na_2_CO_3_. Taurine priming resulted in a greater reduction in the uptake and accumulation of toxic Na. Taurine at 150 mg L^‒1^ exhibited the most significant decrease in leaf Na (26.94%) under Na_2_CO_3_ stress. Conversely, TAR at 200 mg L^‒1^ demonstrated a higher accumulation of root Na (21.82%) under NaCl stress (Figure 3a,b).

A considerable reduction was observed in leaf P (40.48%), root P (43.05%), leaf K (36.38%), root K (37.54%), leaf Ca (30.92%) and root Ca (43.19%) under Na_2_CO_3_ stress compared to the control. Noteworthy enhancement was observed in P, K and Ca content through seed priming with TAR (50, 100, 150, 200 mg L^‒1^). However, TAR at 150 mg L^‒1^ led to a more substantial accumulation of nutrients in both leaves and roots under salinity stress. Leaf phosphorus increased by 10.63%, 21.10%, and 26.22%, while root phosphorus increased by 37.86%, 47.95%, and 23.99% under NaCl, Na₂SO₄, and Na₂CO₃ stress, respectively. Similarly, leaf potassium improved by 31.36%, 63.97%, and 36.97%, and root potassium increased by 19.50%, 45.10%, and 53.09% under NaCl, Na₂CO₃, and Na₂SO₄ stress, respectively. In the case of calcium, leaf calcium content rose by 21.55%, 33.04%, and 29.26%, while root calcium increased by 23.25%, 42.87%, and 37.69% under NaCl, Na₂SO₄, and Na₂CO₃ stress, respectively (Figure C-H).

## Discussion

The rising salinity in agricultural soils is a significant issue, as it adversely affects plant growth and results in substantial yield losses.^58^ Plants experiencing salinity stress exhibit poor growth due to ionic imbalance and the disruption of nutrient uptake.^59^ Nevertheless, using taurine (TAR) in plants under saline conditions can mitigate the adverse effects of chromium and drought, thereby enhancing plant growth and survival.^60^ Our experimental findings demonstrated comparable outcomes, indicating that both saline and alkaline stress conditions resulted in reduced growth of pea plants relative to the control group (Table 1). The presence of salt in the soil impaired water absorption by the seedlings, causing osmotic stress. Additionally, the accumulation of excessive sodium and chloride ions in plant cells inhibited potassium concentration, leading to ionic toxicity.^61^ Research has demonstrated that TAR exhibits the capacity to promote plant growth under abiotic stressed conditions by alleviating oxidative stress.^60^ The reduction in Fv/Fm values indicate stress-induced photoinhibition, while increased NPQ suggests efficient dissipation of excess light energy as heat. These fluorescence metrics correlate with leaf photosynthetic performance, influencing factors such as stomatal conductance and transpiration rate.

Chlorophyll fluorescence parameters effectively indicate the intrinsic connection between salinity stress and photosynthesis.^62^ A higher ΦPSII value signifies more efficient utilization of light energy. Specifically, qP quantifies the openness of ΦPSII reaction centers.^63^ In the current investigation, ΦPSII, qP, qL and Fv/Fm declined with salinity stress, whereas ΦNPQ showed an increase. In accordance with our findings, Zhanwu et al.^64^ reported a decrease in chlorophyll fluorescence, except for non-photochemical quenching (NPQ), in *Leymus chinensis* under saline and alkaline stress conditions. Taurine notably increased the values of ΦPSII, qP, qL, and Fv/Fm under saline and alkaline conditions. A plausible explanation for the influence of TAR application on chlorophyll fluorescence parameters may be attributed to the capacity of TAR to enhance the intensity of light absorption and the light capture efficiency of leaves, potentially due to an increase in leaf area. (Table 2). The application of taurine may modulate ΦPSII activity to efficiently utilize excess light energy and promote Calvin cycle functionality, thereby mitigating damage to the reaction center induced by salinity stress.

Our results demonstrated a significant decrease in leaf relative water content (LRWC) in pea plants subjected to neutral and alkaline toxicity (Table 3). One common consequence of salt toxicity is changes in plant-water relations.^65^ Additionally, neutral and alkaline toxicity induces osmotic stress, possibly reducing LRWC.^66^ According to Shen et al.^67^ oxidative damage significantly reduces the water status of plants under neutral and alkaline salinity conditions. Taurine substantially mitigated oxidative damage, which may have enhanced LRWC in pea plants exposed to salinity stress.

The concentration of chlorophyll pigments decreased as a result of ionic toxicity and osmotic stress induced by salinity, which was attributed to the upregulation of chlorophyllase activity.^68^ Furthermore, we observed a significant increase in oxidative damage due to enhanced ROS generation, which potentially led to the degradation of chlorophyll pigment. In our study, neutral and alkaline salinity reduced the levels of chlorophyll *a*, chlorophyll *b*, total chlorophyll, and carotenoids Taurine notably enhanced these photosynthetic pigments, likely due to decreased production of ROS and reduced sodium uptake (Table 3). These results align with those reported in earlier studies (Ashraf et al.;^69^ Hafeez et al.^24^ in which TAR application restored chlorophyll and carotenoid levels by reducing ROS production under abiotic stress conditions.

β-carotenes contribute to the neutralization of free radicals, thus protecting the photosynthetic apparatus from oxidative damage during photosynthesis.^70^ Our findings indicate that neutral and alkaline salinity significantly reduced β-carotene levels (Table 4). Conversely, the application of TAR considerably increased β-carotene levels under stress, suggesting its protective role in the photosynthetic machinery, this aspect is likely responsible for the improved growth observed in pea plants. β-xanthin and β-cyanin protect plants from stress by neutralizing ROS and detoxifying sodium ions.^71^ These compounds play crucial roles in mitigating the adverse effects of salt stress on plant cells through their potent antioxidant properties. β-Xanthin effectively scavenges reactive oxygen species (ROS), thereby reducing oxidative damage and modulating the plant’s stress-response pathways. Meanwhile, β-Cyanin not only neutralizes ROS but also enhances the activity of stress-responsive enzymes, further strengthening the plant’s defense mechanisms under salinity stress.^72^

Our investigation demonstrated the occurrence of oxidative stress, as evidenced by increased concentrations of MDA, H_2_O_2_, O_2_^•‒^, and RMP, in conjunction with enhanced LOX activity in *Pisum sativum* plants exposed to neutral and alkaline stress conditions (Figure 2). Our findings are consistent with the research conducted by Zhao et al.^73^ where pea plants subjected to alkaline and neutral stress conditions exhibited similar results. In saline-alkaline environments, similar results were noted in oat plants.^74^ The current findings are consistent with those of Ali et al.^75^ which reported increased ROS and MDA levels in tomato plants under salinity stress. Moreover, TAR reduced oxidative damage by diminishing lipid peroxidation, ROS generation, and LOX activity, presumably owing to its antioxidant characteristics.

Plants exposed to salinity tend to accumulate higher GSSG levels alongside a substantial decrease in the GSH:GSSG ratio in response to salinity stress.^76^ The results demonstrated a significant increase in GSH concentrations and a decrease in GSH:GSSG ratio under neutral and alkaline stress conditions. Plants subjected to neutral and alkaline stress failed to sustain the glutathione reductase (GR) activity required to replenish GSH levels. Consequently, this deficiency contributed to heightened ROS generation and oxidative damage. The results corroborate previous findings by Sarkar et al.^77^ on maize and Prajapati et al.^78^ on onion, which documented similar responses to neutral and alkaline stress. To maintain cellular redox homeostasis, plants require a precise equilibrium between the concentrations of GSH and GSSG.^79^ Pea plants exposed to neutral and alkaline stress showed a significant reduction in DPPH radical scavenging activity. Taurine treatment significantly enhanced the overall antioxidant capacity of pea plants, as indicated by elevated DPPH activity. Studies on *Citrus aurantium* (Azeem et al.^80^ *Moringa oleifera* Rehman et al.^81^ and *Triticum aestivum* Meena et al.^82^ have similarly reported reduced DPPH in plants subjected to salts stress.

Nitric oxide plays a crucial role in enabling plants to establish defensive mechanisms when confronted with abiotic stresses.^83^ Taurine-treated stressed pea plants exhibited a marked increase in nitrate reductase (NRA) activity, accompanied by a corresponding enhancement in NO content. Similar findings were also reported by Ullah et al.^84^ in mungbean plants exposed to stress. Nitrate reductase serves as a crucial enzyme in nitrogen metabolism, with the capacity to potentially convert nitrite ions to nitric oxide.^85^ Nitric oxide may attenuate oxidative injury by strengthening the antioxidant system in plants.^86^ A noticeable increase in hydrogen sulfide (H_2_S) levels was observed in pea plants subjected to salinity stress. Hafeez et al.^24^ observed comparable results in Lagenaria siceraria plants under stress conditions. Taurine notably enhanced H_2_S levels in pea plants subjected to saline and alkaline stress.

The elevated levels of phenolics, flavonoids, and ascorbic acid in pea plants mitigate the effects of ROS, thereby reducing oxidative injury.^87^ Taurine exhibited a significant capacity to elevate the endogenous levels of phenolics and flavonoids, potentially amplifying the plant ability to tolerate salt stress. When exposed to alkaline and neutral salts, pea plants showed a substantial increase in the activity of antioxidant enzymes SOD, POD, CAT, and APX. Antioxidant enzyme activities were previously reported to increase in sunflower experiencing salinity stress.^88^ Taurine markedly enhanced the activities of antioxidant enzymes, resulting in minimal oxidative damage in plants (Figure 1).

**Figure 4.**
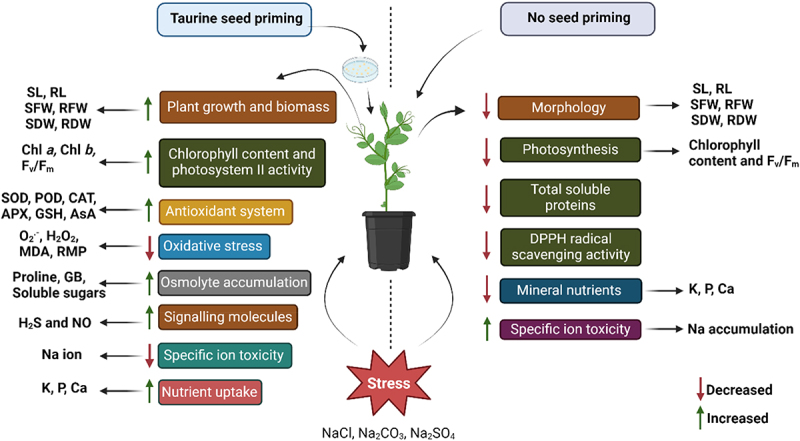
An illustration highlighting the regulation of plant defense responses triggered by taurine seed priming in saline and alkaline environments.

Taurine enhances plant growth by improving the balance between leaf water status and the accumulation of osmolytes.^69^ In this investigation, stressed plants exhibited significantly elevated levels of proline and glycine betaine (GB), indicating their role in reducing water loss in pea plants. Consequently, TAR may contribute to maintaining higher water content in leaves and reducing cellular osmotic potential by promoting the accumulation of these organic solutes. Furthermore, proline and GB are hypothesized to not only facilitate osmotic adjustment under salt stress but also provide protection against the deleterious effects of ROS.^89^ These compounds may contribute to ROS metabolism and maintain membrane integrity. Our findings demonstrated a positive correlation between the production of these organic solutes and a reduction in ROS, which subsequently alleviated stress-induced growth inhibition in pea plants (Table 7). The schematic representation of taurine-mediated regulation of plant defense responses under saline and alkaline conditions is depicted in Figure 4.

**Table 7.** Correlation among Taurine (TAR) application on pea plants under neutral and alkaline stress. Abbreviations: SFW, shoot fresh weight; RFW, root fresh weight; SDW, shoot dry weight; RDW, root dry weight; SL, shoot length; RL, root length; RMP, relative membrane permeability; LRWC, leaf relative water content; NRA, nitrate reductase; GSH, reduced glutathione; GSSG, oxidized glutathione; H_2_O_2_, hydrogen peroxide; MDA, malondialdehyde; Chl *a*, chlorophyll *a*; chl *b*, chlorophyll *b*; total chl, total chlorophyll content; DPPH, 2,2-diphenylpicrylhydrazyl radical scavenging activity; Caro, carotenoids; phenolics; Flavo, favonoids; AsA, ascorbic acid; β-cyanin; β-caro,β-carotenoids; β-xanthin; TSS, total soluble sugars; TSP, total soluble proteins; TFAA, total free amino acids; Proline; H_2_S, hydrogen sulfide; SOD, superoxide dismutase; POD, peroxidase; CAT, catalase; APX, ascorbate peroxidase; LOX, lipoxygenase; non-photochemical quenching of fluorescence; ΦNPQ, the quantum yield of ΦPSП electron transport; FV/FM, photochemical quenching; (qP).

Parameters	Parameters	Correlation coefficient	Parameters	Parameters	Correlation coefficient
**SFW**	T. Chl	0.766***	**LRWC**	RMP	−0.760***
	RMP	−0.754***		H_2_O_2_	−0.735***
	H_2_O_2_	−0.702***		MDA	−0.772***
	MDA	−0.806***		LOX	−0.790***
	Flavonoids	−0.311**		Leaf Na	−0.787***
	Phenolics	−0.356***		Root Na	−0.725***
	Proline	−0.667***		Leaf K	0.614***
	DPPH	0.696***	**H**_**2**_**S**	SOD	0.757***
	ASA	−0.542***		POD	0.711***
	TSP	0.822***		TFAA	0.756***
	TFAA	−0.530***		CAT	0.581***
**T.Chl**	Proline	−0.563***		APX	0.532***
	GSH	−0.312*		LOX	0.745***
	NRA	0.772***		MG	0.642***
	TSS	−0.310**		GB	0.782***
	ASA	−0.524***		TSS	0.619***
	Phenolics	−0.297**	**TSS**	GSH	0.541***
	H_2_S	−0.543***		GSSG	0.430***
	NO	−0.480***		GSH:GSSG	−0.047***
	LOX	−0.790***		H_2_O_2_	0.546***
**Leaf Na**	Leaf P	−0.755***		MDA	0.379***
	Root P	−0.759***		DPPH	−0.410***
	Leaf K	−0.682***		Chl.*a*	−0.278**
	Root K	−0.812***		Chl.*b*	−0.312**
	Leaf Ca	−0.794***	**MDA**	T. Chl	−0.775***
	Root Ca	−0.858***		Caro	0.206*
	H_2_S	0.687***		FV/FM	−0.699***
	LRWC	−0.787***		ΦPSП	−0.665***
	β-cyanin	0.705***		ΦQp	−0.679***
	β -xanthin	−0.518***		QL	−0.535***
	β -caro	0.496***		ΦNPQ	0.545***
	DPPH	−0.857***		NRA	−0.729***

Methylglyoxal (MG) and reactive oxygen species (ROS) play significant roles in plant growth, particularly under stress conditions. MG is a highly reactive compound produced as a byproduct of glycolysis and photosynthesis, which accumulates significantly under abiotic stress, leading to increased oxidative stress through ROS production. This oxidative stress can disrupt cellular functions by causing lipid peroxidation, protein oxidation, and damage to biomembranes.^90^ Salt stress induces oxidative stress by increasing the production of cytotoxic methylglyoxal (MG).^90^ Our findings also showed a significant rise in MG levels under salt stress, suggesting that salinity may impair the activity of enzymes responsible for eliminating MG. However, TAR priming was found to enhance the activity of the MG detoxification system (Gly I and Gly II), leading to a marked reduction in MG concentration in salt-stressed plants. This suggests that TAR may help pea plants better withstand the harmful effects of MG. The protective effect of TAR on stress-induced MG accumulation appears to be linked to its positive impact on glutathione reductase (GR) activity and the regeneration of glutathione (GSH). This aligns with the hypothesis of Guo et al.^2^ who suggested that GSH is key in regulating the glyoxalase system, thus reducing MG content. Similarly, Gupta and Seth^91^ found a positive correlation between the enhanced activity of Gly II and increased GSH levels, as well as the GSH/GSSG ratio (Figure 2).

As shown in Figure 3, this study demonstrates that pea plants pre-treated with TAR experienced a significant improvement in nutrient acquisition under neutral and alkaline salt toxicity. Our results revealed that plants exposed to salt stress had reduced levels of potassium (K), calcium (Ca), and phosphorus (P), while sodium (Na) levels increased. Nutrient acquisition in plants is significantly impaired by saline and alkaline conditions due to the osmotic stress caused by high salt concentrations, which reduces water uptake and disrupts nutrient balance. Under saline conditions, the excessive accumulation of ions like Na^+^ and Cl^−^ competes with essential nutrients such as K^+^, Ca^2 +^, and Mg^2 +^ for uptake sites in roots, leading to deficiencies in these vital minerals. Alkaline conditions can further exacerbate nutrient deficiencies by affecting soil pH, which alters the availability of nutrients. As a result of these interactions between salts and nutrients, plant growth is hindered due to impaired photosynthesis, reduced root development, and increased oxidative stress. The disruption of ion homeostasis led to an accumulation of harmful ions with rise in salinity levels. Jēkabsone et al.^92^ reported that salinity disrupts nutrient balance, causing lower levels of essential nutrient ions and higher Na buildup. The presence of alkaline salts under saline conditions further hindered nutrient uptake by raising pH levels, adding additional stress.^93^ However, TAR priming helped restore nutrient balance and significantly improved the nutrient status of pea plants under both neutral and alkaline salt toxicity.

Taurine supplementation has been demonstrated to enhance nutrient acquisition in plants under saline and alkaline conditions due to its multifaceted role in mitigating stress effects. Taurine functions as an osmoprotectant, facilitating the maintenance of cellular water balance and reducing osmotic stress.^24^ Furthermore, it serves as an antioxidant, scavenging ROS generated under stress conditions, thereby protecting cellular components from oxidative damage. This reduction in oxidative stress contributes to the preservation of cellular membrane integrity and enhances nutrient uptake.^69^ Moreover, taurine modulates ion homeostasis by reducing the accumulation of toxic ions such as sodium (Na^+^) and promoting the uptake of essential nutrients, including potassium (K^+^), calcium (Ca^2 +^), and phosphorus (P). By alleviating both osmotic and oxidative stress, taurine enables plants to absorb and utilize nutrients more effectively, even in challenging environmental conditions.

## Conclusion

In conclusion, salinity stress induced by NaCl, Na_2_CO_3_, and Na_2_SO_4_ significantly reduced various growth attributes, including shoot and root length, fresh and dry weight, and photosynthetic parameters in pea plants. However, seed priming with taurine (TAR) notably alleviated these detrimental effects, improving growth characteristics, chlorophyll fluorescence, and photosynthetic pigment content. TAR (150 mg L^‒1^) was particularly effective, enhancing growth parameters, leaf area, and photosynthetic efficiency under salt stress. Furthermore, TAR increased the accumulation of antioxidants, osmolytes, and essential nutrients, while reducing oxidative stress markers and improving nutrient uptake, especially in terms of phosphorus, potassium, and calcium. These findings suggest that TAR priming has a significant potential to mitigate the adverse effects of salinity, improving plant growth and stress tolerance.

## Abbreviations

ROS, reactive oxygen species; TAR, taurine; Cl^−^, chloride; Na^+^, sodium; H_2_O_2_, hydrogen peroxide; O_2_^•‒^, superoxide radical; MG, methylglyoxal; NaCl, sodium chloride; Na_2_SO_4_, sodium sulfate; Na_2_CO_3_, sodium carbonate; •OH, hydroxyl radical; SOD, superoxide dismutase; CAT, catalase; APX, ascorbate peroxidase; GSH, glutathione; GSSG, oxidized glutathione; LOX, lipoxygenase; MDA, malondialdehyde; H_2_S, hydrogen sulfide; NO, nitric oxide.
